# Leaf Dry Matter Content Predicts Herbivore Productivity, but Its Functional Diversity Is Positively Related to Resilience in Grasslands

**DOI:** 10.1371/journal.pone.0101876

**Published:** 2014-07-08

**Authors:** Robin J. Pakeman

**Affiliations:** The James Hutton Institute, Craigiebuckler, Aberdeen, United Kingdom; Agroecological Institute, China

## Abstract

This paper addresses whether the ecosystem service of animal production from grasslands depends upon plant functional identity, plant functional diversity or if the resilience of production is a function of this diversity. Using the results of nine grazing experiments the paper shows that productivity is highly dependent on one leaf trait, leaf dry matter content, as well as rainfall. Animal (secondary) productivity is not dependent on plant functional diversity, but the variability in productivity of grasslands is related to the functional diversity of leaf dry matter content. This and a range of independent studies have shown that functional diversity is reduced at high levels of grassland productivity, so it appears that there is a trade-off between productivity and the resilience of productivity in the face of environmental variation.

## Introduction

Many ecosystem services are directly dependent on ecosystem processes that are in turn dependent upon the nature of the vegetation present [1]. In order to manage ecosystems to maintain the provision of ecosystem services for the long-term, we need to understand how various characteristics of plant communities contribute to controlling ecosystem processes. This approach has been formalized into analyzing the relative contributions of abiotic factors, plant traits and plant functional diversity to the rate of the process in question [2]. Whilst many studies have shown the dependence of processes such as primary productivity and decomposition on plant traits [3]–[5], there has been less success in identifying the positive role of functional diversity in controlling ecosystem processes [6]–[8] with even negative relationships having been found [9]. This has, perhaps, been hampered by the search for suitable metrics of functional diversity [10].

Plant functional diversity could contribute to ecosystem processes in two ways. Functional diversity could contribute directly to the process rate [2], [11] (Hypothesis 1) or it could contribute to the resilience of ecosystem service supply, in this case animal production (Hypothesis 2) – where resilience is defined as the capacity of a system to deliver services in the face of disturbance [12], [13], or in other words, reduce the variability of the process in question [14]. Resilience is necessary to sustain desirable ecosystem states in variable environments and uncertain futures [15]. However, as functional diversity is sensitive to land use intensification [16] then understanding its contribution to ecosystem services is a necessary step to enable the future long-term provision of services.

Many studies use ecosystem processes as surrogates for ecosystem services. This study instead, took a direct measure of an ecosystem service - livestock productivity from grasslands - measured as the density of livestock that can be stocked to achieve a set vegetation height. The data were derived from nine long-term grazing experiments [17] which had simultaneous measures of grazing density and vegetation data. Using weather data and trait data from databases, the influence of growing season weather, plant traits (as their community weighted mean) and plant functional diversity on production were tested. Functional diversity was calculated for a suite of response traits [5] and for individual leaf traits known to be linked to ecosystem function [18], [19]. There is no consensus on which measures of functional diversity may drive ecosystem function/services. Many functional metrics are highly correlated [18], so to minimize the number of variables tested to reduce the risk of type one errors, only two orthogonal metrics were calculated: Rao's entropy (Q), which combines both functional richness and divergence, and the functional evenness (FEve) [20]. Two alternate hypotheses were tested: (1) that functional diversity contributes directly to ecosystem service delivery, and (2) that functional diversity confers resilience (the capacity of the system to deliver the service in the face of changing conditions) to ecosystem services through the presence of alternative trait combinations that can exploit the variance in environmental conditions.

## Material and Methods

The results of nine previously published experiments investigating the interactions between livestock grazing and the dynamics of grass-dominated vegetation were assembled. These were the same as those analyzed in [17], except for one experiment (Bell Hill, [21]) for which the management data were not complete. Data were available for between 4 and 15 years depending on experiment and consisted of vegetation records (point quadrat data) and productivity data in terms of the number of days of grazing required to keep the vegetation at a set height - this equates to secondary production. Data were taken just from the control (continuation of previous site management) treatments so the data were not confounded by changing grazing intensities and were converted to livestock units (LU) ha^−1^ yr^−1^ to account for the different species of grazers in the different experiments [22]. The data are available from the author. Trait data were assembled from standard sources [23], [24] but were restricted to the main response traits in grassland vegetation [5] (Table 1). Data for numeric traits were usually averaged from many entries in the databases; mean values for each species were calculated by a weighted mean of all the entries, where the weight was represented by the replication used to produce each entry. Using trait information sourced from databases meant that species values were fixed both within and between sites, so changes in the values of numeric traits between years depends upon changes in abundance only. The trait and vegetation data were used to calculate the following functional diversity measures for the full set of response traits and for the two individual leaf traits SLA and LDMC: community weighted mean (CWM), and two orthogonal measures of functional diversity - functional evenness (FEve) and Rao's Q [16]. The two vegetative regeneration attributes were each given a weight of 0.5 to ensure the overall weight of this trait was the same as for the others. Weather data were assembled from the UKCP09 5 km×5 km gridded data available for each month [25]. For each site the growing season's mean temperature and total rainfall were calculated for the period May to September, as this was the period over which the grazing ran for all experiments.

**Table 1:** Traits used in the analysis with source and coding information.

Traits	Coding	Attributes
Bud height (life-form) ^(*)^	0	Geophyte, Therophyte
	0.333	Hemicryptophyte
	0.667	Chamaephyte
	1	Phanerophyte
log Canopy height (m) ^(†)^	continuous	
Canopy structure^(*)^	0	Rosette
	0.5	Hemirosette
	1	Erosulate
Flowering - start (month) ^(*)^	1–12	
Leaf Dry Matter Content (mg g^−1^) ^(†)^	continuous	
log Leaf size (mm^2^) ^(†)^	continuous	
^1^Leafing period - summer green^(*)^	0	Evergreen
	1	Summer green
Life-span^(*)^	0	Annual
	0.5	Biennial
	1	Perennial
Specific Leaf Area (mm^2^ mg^−1^) ^(†)^	continuous	
Vegetative spread - rhizome^(*)^	0	Not rhizomatous
	1	Rhizomatous
Vegetative spread - stolon^(*)^	0	Not stoloniferous
	1	Stoloniferous

Sources of data: *BiolFlor [33], ^†^LEDA [32].

Assuming that productivity is a function of plant response traits and weather (Hypothesis 0), the analysis was set up to address the following hypotheses: (1) that functional diversity can help explain the variation in productivity, and (2) that functional diversity is correlated to the resilience of site productivity. Hypothesis 0 corresponds to following stages 1 and 2 of (2) and then linking them together as it is already understood that productivity is frequently dependent on both weather [26] and on the functional characteristics of the vegetation [4]. Hypothesis 1 follows their steps 3 and 5: identifying possible functional diversity measures related to productivity and then the most parsimonious model of productivity from the combination of trait, weather and functional diversity previously identified as correlated to the ecosystem process of interest.

**Figure 1:**
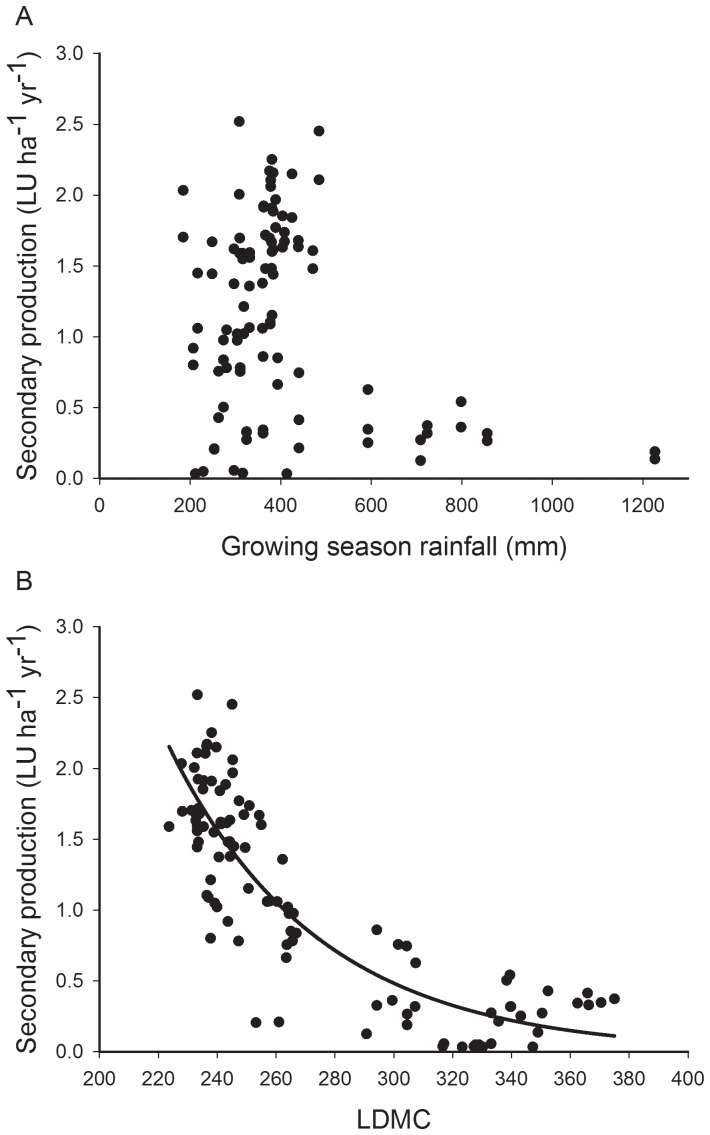
Productivity relationships. Fitted relationships between (A) rainfall (mm) and (B) leaf dry matter content (LDMC, mg g^−1^) and production (LU ha^−1^ yr^−1^).

Developing the models through simplification from a full model was not possible due to the number of potential alternatives. Instead, Hypothesis 0 was addressed by finding the combination of weather and traits that best explained the productivity of the vegetation starting from a fixed model of the best response trait (this was highly correlated to the other significant response traits 0.554 to 0.946, and fitting them subsequently to the best leaf trait did not offer significant explanatory power) and all the climate variables [27], [28]. A squared term was added for yearly rainfall as a result of data exploration. Leaf dry matter content (Fig. 1b) showed an exponential decay. This was transformed using a non-standard transformation (e^−0.01704LDMC^), determined by fitting a non-linear regression using *nls* in R, after initial parameter estimation using *SSaymp* [29]. The random model was simplified first [28], starting from date|plot +1|experiment/block/plot, by comparing the impact of term removal on AIC. The eventual random model used was 1|experiment/plot. The fixed model was then simplified by comparing likelihood ratios and AIC values from progressive removal of terms. Models were fitted using the *nmle* package [30] in *R* (R Development Core Team 2010) using maximum likelihood for fixed model comparisons, and residual maximum likelihood for random model comparisons and final estimations of model parameters. Finally, any temporal autocorrelation of the final model was assessed using the *ACF* function [29]. There was no significant temporal autocorrelation; productivity was not dependent upon the previous growing season's weather.

Having identified the best model for Hypothesis 0, further models to test Hypothesis 1 were built with forward selection on the same random model. The possible additional fixed effects were the two overall measures of functional diversity calculated from the response traits in Table 1 and the two measures calculated for the identified trait in Hypothesis 0; LDMC. Parameter additions were tested through likelihood ratios from models identified with maximum likelihood, and residual maximum likelihood used for final estimations of model parameters [28]. In addition, the mean across years of the functional diversity measures and productivity per plot were correlated to see if long-term grazing patterns impacted functional diversity (using a linear mixed model with a random model of 1|experiment).

Hypothesis (2) was addressed by taking the absolute values of the raw residuals from the combined trait and climate model from Hypothesis 0 and testing to see if there was a relationship between them and the functional diversity measures tested in Hypothesis (1). This, in effect, tested whether functional diversity was correlated to the divergence from expectation, with the expectation that the higher the functional diversity the lower the divergence – which can be seen as a measure of resilience. The random model was the same as for the other two hypotheses. However, as this analysis used the output of a previous analysis, the degrees of freedom were revised downwards by one reflect the number of previously fitted parameters and hence prevent an overly liberal test [31].

## Results

There was a weak curvilinear relationship between animal productivity and growing season rainfall indicating limitations at both high and low rainfall (Table 2, Fig. 1a). There was no significant relationship with temperature. Leaf Dry Matter Content (LDMC) was the most successful leaf trait predictor of production (Fig. 1b), though some other non-leaf traits were also well correlated including life-form and vegetative spread (Table S1). The relationship was a clear exponential decay, with very low animal productivity at high values of LDMC. SLA did not have a significant relationship with productivity. The best climate and trait model was a combination of LDMC and a linear term for rainfall; the relationship with rainfall was positive suggesting drought limitation of productivity (Table 2; Table S2).

**Table 2:** Model parameters, parameter probabilities and model fits for the best models containing weather variables, trait variables and the combined models of traits and weather.

Parameter sources	Fitted relationship	df	p-value
Weather only	0.229	88	0.370
	+0.00234YearRain		<0.001
	−1.47×10^−6^Rain^2^		0.003
Trait only	0.240	81	0.179
	+84.80×e^−0.0170LDMC^		<0.001
Trait + weather	−0.687	80	<0.001
	+95.31×−e^−0.0170LDMC^		<0.001
	+0.0018YearRain		<0.001

LDMC leaf dry matter content, YearRain rainfall (mm) during the growing season – 1 May to 30 September.

**Figure 2:**
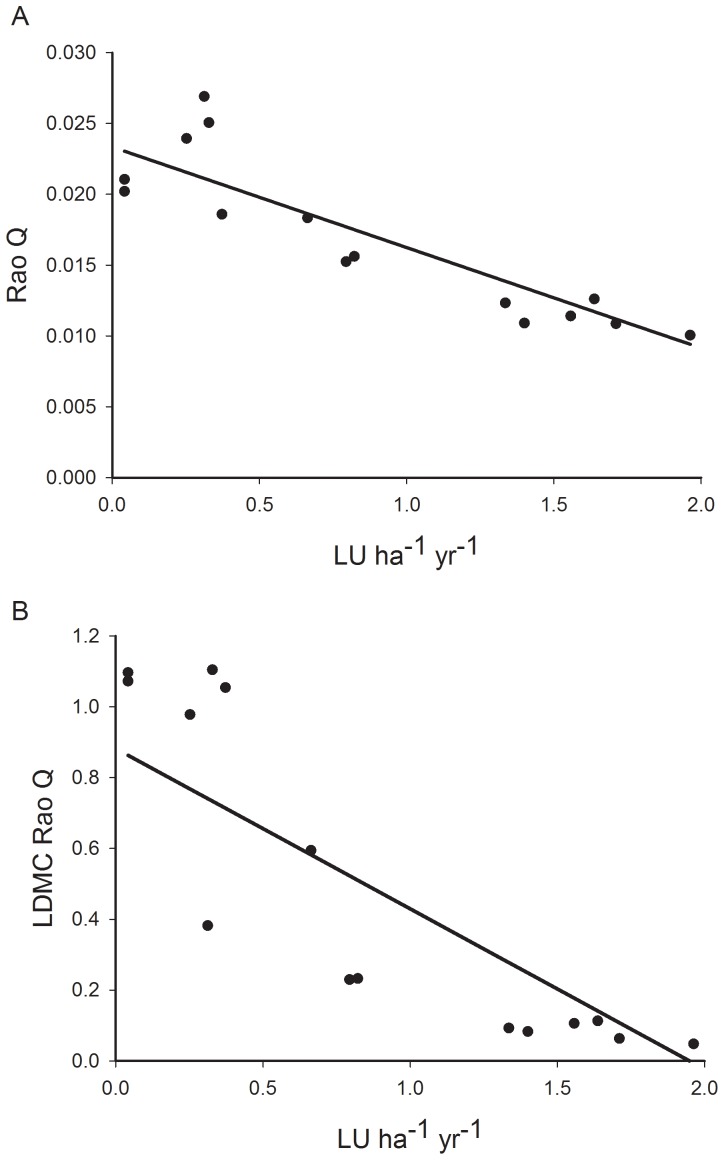
Productivity:functional diversity relationships. Fitted relationships between plot mean production ((LU ha^−1^ yr^−1^) and (A) mean plot Rao's Q and (B) mean plot LDMC Rao's Q.

This combined weather and trait model was taken as the baseline for judging how functional diversity affects production. However, none of the four possible, additional predictors (overall and LDMC measures of FEve and Rao's Q) could be added to this model based on the presence of significant additional terms (Hypothesis 1, p from 0.078 to 0.709, Table S3), though two measures, overall Rao's Q and LDMC FEve, gave a small reduction in AIC. Despite the functional diversity measures not improving the model from Hypothesis 0, there were clear relationships between mean productivity and mean overall Rao's Q (p = 0.005, Fig. 2a) and with mean LDMC Rao's Q (p = 0.017, Fig. 2b).

**Figure 3:**
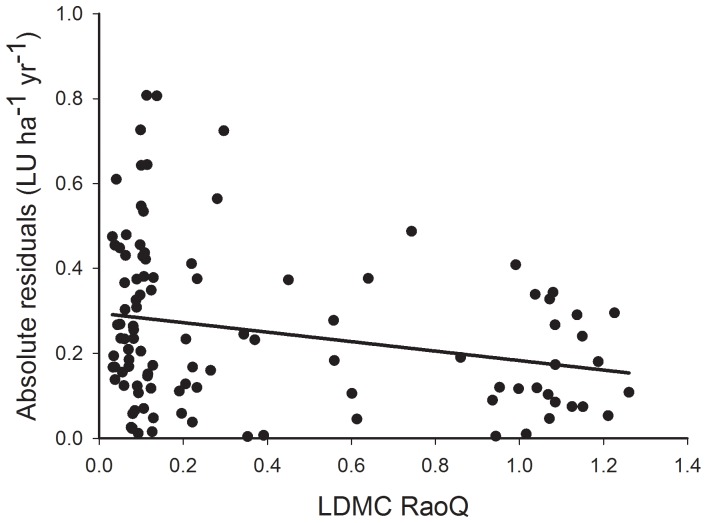
Resilience and functional diversity. The response of the absolute residual after fitting LDMC and rainfall to the Rao's Q of LDMC. Details of model fit and parameters are in Table 2. Details of the LDMC and rainfall model are given in Table 1.

The alternative approach of analyzing for correlates of the absolute residuals from the trait and weather model (Hypothesis 2) showed that functional diversity, as Rao's Q of LDMC, was negatively correlated to the size of the residuals (Fig. 3); variability is reduced at high functional diversity. However, the strength of the relationship was relatively weak (p = 0.042), though significant (Table 3). Neither metric of overall functional diversity were helpful in explaining the residuals (p = 0.459, 0.869 respectively), nor was functional evenness of LDMC (p = 0.983).

**Table 3:** Model parameters, parameter probabilities and model fit for relationships between functional evenness (FEve) and Rao's Q from the full trait list and for LDMC alone and the absolute residuals from the combined trait and climate model described in Table 2.

Functional diversity measure	Fitted relationship	Adjusted df	p-value
FEve	0.299	79	<0.001
	−0.108 FEve		0.459
Rao's Q	0.251	79	<0.001
	−0.728 RaoQ		0.869
LDMC FEve	0.241	79	<0.001
	−0.003 LDMCFEve		0.983
LDMC Rao's Q	0.278	79	<0.001
	−0.104 LDMCRaoQ		0.043

## Discussion

A single leaf trait, community weighted LDMC, proved to be a robust predictor of ecosystem productivity. The choice of LDMC over SLA as a measure of productivity is in contrast to many studies [4], [32], but aligns with some [18], [33]. LDMC is a good measure of investment in leaf structural tissue and its inverse is hence a good measure of digestibility [34]. Substituting LDMC by other traits such as leaf nitrogen content could be even more effective. However, the availability of this data was not as complete as for LDMC for this analysis. Identifying good predictors of ecosystem processes and services, such as here, provides the opportunity to map ecosystem services based on vegetation information [1], for instance to highlight areas of high value, and to analyze the trade-offs in service supply that would occur as a result of land use change [35]. As LDMC appears sensitive to average rainfall at both a specific [36] and community level [37], the small additional explanatory power that rainfall makes to a model fitted with LDMC alone is as expected. Rainfall here explained inter-annual variation in production whilst LDMC is explained by the climate (as well as soil) driven variation.

Functional diversity was not helpful in explaining the productivity of the grasslands analyzed. Thus this study does not add evidence either way to the role of functional diversity in contributing to productivity [7], [38] or restricting it [9]. The marginally significant negative relationships (Table S2) were, however, more in line with the latter – as with an increase in the variance of traits present there has to be an increase in the absolute distances between species traits present and the to the optimum trait value for those specific conditions of site and weather combined. In line with other studies [39], [40], functional diversity was reduced at high land use intensity, as was the functional diversity of leaf dry matter content. However, here there was evidence that functional diversity, as the richness/divergence measure Rao's Q of LDMC, was correlated to the size of the residuals from the model containing LDMC and rainfall. At high functional diversity the grasslands appeared to be more statistically predictable in their productivity, suggesting a higher resilience to environmental variability. This may explain the patterns seen in other studies where species diversity was correlated to resilience in production [41]; species diversity was likely a surrogate for functional diversity. However, meta-analysis has shown that the stability of service delivery varies between the type of stressor, and the pattern shown here in response to weather may not be similar in response to other disturbances [42] The relationship identified here was relatively weak, but it was present after accounting for the fitting of LDMC and rainfall, and a range of other factors will have contributed to the unexplained variation, including intra-annual variability in the weather.

From the data analyzed here it appears that functional diversity, a key component of biodiversity, is contributing to the sustainability of grassland production systems through increased resilience and predictability of outputs. Managing for high production may result in reduced the statistical predictability of production [43] as well as reducing biodiversity [44], [45]. Consequently, there appears to be a trade-off between productivity and resilience of production that is mediated via the functional diversity and functional identity of the plant community. This is in addition to the well-known impact of increase agricultural production on biodiversity.

## Supporting Information

Table S1 **Productivity correlations with non-leaf traits.** Model parameters, parameter probabilities and model fits for alternative models linking traits to production.
(DOCX)

Table S2 **Stages in productivity model simplification.** Random and fixed model parameters in the stages of model simplification [28].
(DOCX)

Table S3 **Models combining productivity and functional diversity.** Tests of adding functional diversity parameters to the trait and climate model using maximum likelihood and a likelihood ratio statistic to assess their additional explanatory power.
(DOCX)
