# Supplementary material for: Leaf Dry Matter Content Predicts Herbivore Productivity, but Its Functional Diversity Is Positively Related to Resilience in Grasslands
Source: PLoS One. 2014 Jul 8;9(7):e101876. doi: 10.1371/journal.pone.0101876 (PMC4086977; doi:10.1371/journal.pone.0101876)
Supplement: Table S1 — Productivity correlations with non-leaf traits. Model parameters, parameter probabilities and model fits for alternative models linking traits to production. (DOCX) [file pone.0101876.s001.docx]

**Table S1.** Model parameters, parameter probabilities and model fits for alternative models linking traits to production.

| Parameter sources | Fitted relationship | df | p-value |
| --- | --- | --- | --- |
| Bud height | 4.095 | 89 | <0.001 |
|  | -9.242BudHt |  | <0.001 |
| Rhizome | 1.581 | 89 | <0.001 |
|  | -2.017Rhizome |  | <0.001 |
| Leaf Size | 0.468 | 89 | <0.001 |
|  | +0.002LeafSize |  | <0.001 |

Bud height and rhizome were weighted proportions and hence range from 0 to 1, for bud height a trait value of 0 represents geophytes and annuals, whilst 1 represents phanerophytes (Table 1). Leaf size (mm^2^) was log-transformed prior to calculation of the community weighted mean.
