# Supplementary material for: Leaf Dry Matter Content Predicts Herbivore Productivity, but Its Functional Diversity Is Positively Related to Resilience in Grasslands
Source: PLoS One. 2014 Jul 8;9(7):e101876. doi: 10.1371/journal.pone.0101876 (PMC4086977; doi:10.1371/journal.pone.0101876)
Supplement: Table S2 — Stages in productivity model simplification. Random and fixed model parameters in the stages of model simplification [28]. (DOCX) [file pone.0101876.s002.docx]

**Table S2.** Random and fixed model parameters in the stages of model simplification [28].

| Random and fixed parameters | AIC | Likelihood ratio | p-value |
| --- | --- | --- | --- |
| Random model reduction |  |  |  |
| Date\|Plot + 1\|Experiment\Block\Plot | 119.99 |  |  |
| 1\|Experiment\Block\Plot | 107.99 |  |  |
| 1\|Experiment\Plot | 105.99 |  |  |
| Fixed model reduction |  |  |  |
| e^-0.0170LDMC^+YearRain +YearRain^2^+YearTemp | 65.64 |  |  |
| e^-0.0170LDMC^+YearRain +YearRain^2^ | 65.13 | 1.49 | 0.222 |
| e^-0.0170LDMC^+YearRain | 65.02 | 2.07 | 0.150 |

Fixed model reduction used maximum likelihood, and hence resulted in different AIC values. LDMC leaf dry matter content, YearRain rainfall (mm) during the growing season – 1 May to 30 September. YearTemp mean temperature (°C) through the growing season.
