# Supplementary material for: Leaf Dry Matter Content Predicts Herbivore Productivity, but Its Functional Diversity Is Positively Related to Resilience in Grasslands
Source: PLoS One. 2014 Jul 8;9(7):e101876. doi: 10.1371/journal.pone.0101876 (PMC4086977; doi:10.1371/journal.pone.0101876)
Supplement: Table S3 — Models combining productivity and functional diversity. Tests of adding functional diversity parameters to the trait and climate model using maximum likelihood and a likelihood ratio statistic to assess their additional explanatory power. (DOCX) [file pone.0101876.s003.docx]

**Table S3.** Tests of adding functional diversity parameters to the trait and climate model using maximum likelihood and a likelihood ratio statistic to assess their additional explanatory power.

| Fixed model | AIC | Likelihood ratio | p-value |
| --- | --- | --- | --- |
| e^-0.0170LDMC^+YearRain | 65.02 |  |  |
| e^-0.0170LDMC^+YearRain + FEve | 67.07 | 0.14 | 0.709 |
| e^-0.0170LDMC^+YearRain + Rao’s Q | 64.10 | 3.10 | 0.078 |
| e^-0.0170LDMC^+YearRain + LDMC FEve | 64.47 | 2.74 | 0.098 |
| e^-0.0170LDMC^+YearRain + LDMC Rao’s Q | 65.17 | 2.03 | 0.154 |
